# Supplementary material for: Double drives and private alleles for localised population genetic control
Source: PLoS Genet. 2021 Mar 23;17(3):e1009333. doi: 10.1371/journal.pgen.1009333 (PMC8018619; doi:10.1371/journal.pgen.1009333)
Supplement: S1 Text — (PDF) [file pgen.1009333.s001.pdf]

## Supplemental methods.

### *Genetics*

Our baseline model has 2 autosomal loci each with 3 alleles. At the first locus A is the wildtype allele; $\alpha$ is the transgenic construct inserted into and disrupting the wild type allele; and a is a cleavage-resistant allele produced by end-joining repair. For population suppression we assume that the A locus is needed for female survival past the pupal stage, that the resistant allele is non-functional, and that functional resistance is not possible [1]. When A is a haplo-sufficient gene, we assume no reduction in survival probability when carrying only one functional copy of the gene. At the second locus there are initially 2 segregating alleles of equal fitness, B and b, with B being more common in the target population than in the non-target population (and vice versa for b). The third allele, $\beta$, is the transgenic construct which can home into B but not into b. Where the differentiated B locus is neutral, we assume all end-joining repair events produce cleavage-resistant alleles (b). In the case where the differentiated B locus is an essential gene, we extend the model to have a 4th allele at this locus, bj, which is a non-functional, cleavage resistant, version of B that can be formed by end-joining repair and assume all end-joining leads to non-functional alleles. We also assume that the $\beta$ construct has been engineered to have minimal effects on fitness (e.g., by having a recoded version of the B allele, or being inserted into an artificial intron). For comparison we also model a single drive consisting of a single construct homing into a female viability gene, and allow for both functional resistance genes that may pre-exist in the population and non-functional resistance alleles created by end-joining (4 allele model; Fig 3 in main text).

For population replacement where $\alpha$ carries a desirable cargo gene and $\beta$ is in the differentiated locus, we consider three cases, where (i) both A and B loci are selectively neutral; (ii) the A locus is selectively neutral and B is a haplo-insufficient essential gene; or (iii) the A locus is a haplo-insufficient essential gene and the B locus is selectively neutral. In those cases where the A or B locus is an essential gene, the $\alpha$ or $\beta$ construct is assumed to have been designed to have minimal effects on fitness (as above), end-joining produces non-

functional alleles which are dominant lethal, and any pre-existing resistant allele (at B) is selectively neutral. In each case there are 3 alleles at each locus, except when B is an essential gene, when there are 4. For comparison we also consider a single drive consisting of a construct that homes into a neutral site (3 allele model) or into a haplo-insufficient essential gene (4 allele model; Fig 6 in main text).

***Fitness effects of disrupting host genes***

The fitness of a genotype depends both on the disruption of host genes caused by insertions and mutations and on the activity of the construct(s) it carries. For the fitness effects due to disruption of host genes the fitness of A/A and B/B homozygotes is standardised to 1, and the fitness of genotypes homozygous for the construct $\alpha/\alpha$ or β/β is $1 - s_I$; homozygous for the cleavage resistant allele a/a or b/b is $1 - s_R$; heterozygous for the construct A/$\alpha$ or B/β is $1 - h_I s_I$; heterozygous for the cleavage resistant allele A/a or B/b is $1 - h_R s_R$ or heterozygous carrying both the construct and cleavage-resistant allele $\alpha$/a or β/b is $1 - s_{IR}$, where $s_I$, $s_R$ and $s_{IR}$ are selection coefficients and $h_I$ and $h_R$ are dominance coefficients, each of which differ depending on the locus (A and B) and between sexes (see S2 Table for more details of locus-specific fitness costs for each scenario modelled).

***Construct activity and fitness effects***

Each construct consists of one or more transcription units (Cas9, gRNA, X-shredder, Cargo), and the activity associated with each is assumed to be dominant (i.e., dosage independent). For genotypes carrying at least one Cas9, one gRNA and the site targeted by the gRNA (A or B), cleavage of the target site occurs with probability $c$, after which end-joining repair occurs with probability $j$ converting the target site to a resistant allele (a or b), and homing occurs with probability $1 - j$ converting the target site to the construct ($\alpha$ or β). For males carrying at least one X-shredder, sperm is produced carrying Y or X chromosomes at a ratio of $m{:}\,1 - m$. In all models, we assume a fitness cost due to off-target cleavage ($sH$) of 1%, where fitness is reduced by a factor $1 - sH$ if there is at least one Cas9 and one gRNA present, or by a factor $(1 - sH)^2$ if there is at least one Cas9 and two different gRNAs present.

To model costs due to somatic expression of the nuclease, genotypes carrying at least one Cas9, one gRNA and the site targeted by the gRNA, have fitness reduced by a factor $1 - sS$. Similarly, to model costs associated with X-shredding, males carrying at least one X-shredder have fitness reduced by a factor $1 - sX$. With the exception of Fig 5B and C in the main text, we assume both $sS$ and $sX$ to be zero. For population replacement we assume the cargo has a fitness cost ($sC$) of 20% in females, with no effect on males. In all cases the fitness costs associated with each unit's activity are assumed to be dominant (i.e., dosage independent), and for simplicity, are assumed to affect survival after density dependence and before censusing (e.g., at the pupal stage).

The overall fitness of a genotype is the product of the relative fitness due to host gene disruption at locus A, host gene disruption at locus B and construct activity.

***Analyses of evolutionary stability***

We extend both the 3-allele and 4-allele models to allow for loss-of-function mutations of each transcription unit (Cas9, gRNA, X-shredder, cargo). After release of fully functional constructs, we assume that each unit mutates with probability $\mu$; mutation can only occur during homing; it is possible for more than one unit to mutate during a single homing event; and mutation back to a functional unit does not occur. Constructs carrying non-functional units are expected to carry the fitness costs associated with disrupting the function of the host gene but no longer carry costs due to the transcription unit activity (e.g., off-target cleavage). Although at the sequence level constructs carrying only non-functional units are expected to differ from cleavage-resistant alleles produced through end-joining repair, both are assumed to be resistant to cleavage and carry the same fitness costs, and we model them as a single allele.

***Population biology***

We model a population with discrete, non-overlapping generations. In each generation, we assume the population mates randomly, that females produce $f$ fertilised eggs and that males are not limiting in their production. Juvenile survival is density-dependent, such that the

probability of surviving is $\theta_J \frac{\gamma}{\gamma + N_J}$, where $\theta_J$ is the density-independent probability the juvenile survives to adulthood, $\gamma$ determines the strength of density dependent mortality and $N_J$ is the number of juveniles in the population. All our results are in terms of relative population sizes (compared to the pre-release equilibrium), and are unaffected by the precise value of $\gamma$. The intrinsic rate of increase ($R_m$) of the wild-type population is $\frac{f\theta_J}{2}$. After density-dependent juvenile mortality, additional genotype-dependent mortality occurs before juveniles mature into adults, taking into account fitness costs associated with the constructs and cleavage-resistant alleles. Adult males and females then produce gametes, during which recombination occurs between the A and B loci with probability $r$, and, depending on the genotype, cleavage followed by homing or mutation may occur in males or females, and shredding of the X-chromosome may occur in males.

***Population censusing***

Relative population densities and allele frequencies are of populations censused at the adult stage. The correlation between the constructs $\alpha$ and $\beta$ is calculated as:

$$\frac{(p_{\alpha\beta} - p_\alpha * p_\beta)}{\sqrt{p_\alpha(1-p_\alpha)p_\beta(1-p_\beta)}} \quad (1)$$

where, $p_{\alpha\beta}$ is the frequency of $\alpha\beta$ chromosomes in the population and $p_\alpha$ and $p_\beta$ are the frequency of $\alpha$ and $\beta$ alleles in the population respectively.

***PAM site analysis in An. gambiae***

To identify PAM sites (NGG or CCN) in *An. gambiae* with the type of differentiion required for our approach, SNP variants from the Phase II Ag1000G dataset were first screened for the presence of at least one G or C allele. Positions upstream and downstream of the variant were then screened to calculate frequencies of GG and CC dinucleotides, using the AgamP4 reference genome for invariant neighbouring sites, or the Ag1000G SNP data for neighbouring variants. Since phased data was only available for biallelic variants, dinucleotides containing two variants, of which at least one was multiallelic, were not screened (24% of G or C variants). For each differentiated PAM site, frequencies were calculated for

the 16 sampled populations (Cameroon *An. gambiae* n=594, Uganda *An. gambiae* n=224, Burkina Faso *An. gambiae* n=184, Guinea-Bissau uncertain sp. n=182, Angola *An. coluzzii* n=156, Burkina Faso *An. coluzzii* n=150, Cote d'Ivoire *An. coluzzii* n=142, Gabon *An. gambiae* n=138, The Gambia uncertain sp. n=130, Ghana *An. coluzzii* n=110, Kenya uncertain sp. n=96, Guinea *An. gambiae* n=80, Mayotte *An. gambiae* n=48, Ghana *An. gambiae* n=24, Bioko *An. gambiae* n=18 and Guinea *An coluzzii* n=8, where n is the number of sequences and n/2 is the number of individuals sampled). We included populations of *An. coluzzii* and of uncertain species assignment because there can be frequent hybridisation with *An. gambiae*. PAM sites containing >5% missing data at any sampling location were removed (0.5% PAMs), leaving a total of 13,462,450 polymorphic PAM sites. The per-site PAM frequencies were used to identify PAM sites present in the island population of interest, and at frequencies <10%, <5% or absent in all other populations, excluding the *An. coluzzii* population from Guinea due to low sample size (n=8, from 4 individuals). Analysis of the Ag1000G data made heavy use of the python package scikit-allel (https://doi.org/10.5281/zenodo.3238280).

## References


1. Kyrou K, Hammond AM, Galizi R, Kranjc N, Burt A, Beaghton AK, et al. A CRISPR-Cas9 gene drive targeting *doublesex* causes complete population suppression in caged *Anopheles gambiae* mosquitoes. Nat Biotechnol. 2018;36(11):1062-6. Epub 2018/09/25. doi: 10.1038/nbt.4245. PubMed PMID: 30247490.
