# Supplementary material for: Double drives and private alleles for localised population genetic control
Source: PLoS Genet. 2021 Mar 23;17(3):e1009333. doi: 10.1371/journal.pgen.1009333 (PMC8018619; doi:10.1371/journal.pgen.1009333)
Supplement: S1 Table — (PDF) [file pgen.1009333.s002.pdf]

Parameter	Description	Baseline value
R_m	Intrinsic rate of population increase	6
r	Recombination between loci	0.5
d	Proportion of offspring carrying transgene	0.976 [1]
u	Proportion of non-homed chromosomes which are mutant	0.392 [2]
e	Probability of homing	$2\mathrm{d} - 1 = 0.953$
c	Probability of cleavage	$e + (1 - e)\mathrm{u} = 0.971$
j	Probability of end joining given cleavage	$\frac{(1-e)u}{e+(1-e)u} = 0.019$
m	Proportion of Y-bearing sperm produced by X-shredder males	0.95 [3]
s_H	Cost of off-target cleavage in individuals expressing Cas9 and gRNA (targeting either allele A or B) (dominant)	0.01
s_S	Female cost of somatic cleavage of wildtype allele by Cas9 and gRNA (targeting either allele A or B) (dominant)	0
s_X	Male cost of X-shredding (dominant)	0
s_C	Female cost of cargo expression	0.2
p	Probability of a functional mutant being produced through end joining	0
μ	Probability of each component becoming non-functional during homing	0

References

1. Kyrou K, Hammond AM, Galizi R, Kranjc N, Burt A, Beaghton AK, et al. A CRISPR-Cas9 gene drive targeting *doublesex* causes complete population suppression in caged *Anopheles gambiae* mosquitoes. Nat Biotechnol. 2018;36(11):1062-6. Epub 2018/09/25. doi: 10.1038/nbt.4245. PubMed PMID: 30247490.

2. Hammond A, Karlsson X, Morianou I, Kyrou K, Beaghton A, Gribble M, et al. Regulating the expression of gene drives is key to increasing their invasive potential

and the mitigation of resistance. PLOS Genet. 2021;17(1):e1009321. doi: 10.1371/journal.pgen.1009321.

3. Galizi R, Doyle LA, Menichelli M, Bernardini F, Deredec A, Burt A, et al. A synthetic sex ratio distortion system for the control of the human malaria mosquito. Nat Commun. 2014;5:3977. Epub 2014/06/11. doi: 10.1038/ncomms4977. PubMed PMID: 24915045; PubMed Central PMCID: PMCPMC4057611.
