# Supplementary material for: Double drives and private alleles for localised population genetic control
Source: PLoS Genet. 2021 Mar 23;17(3):e1009333. doi: 10.1371/journal.pgen.1009333 (PMC8018619; doi:10.1371/journal.pgen.1009333)
Supplement: S2 Table — (PDF) [file pgen.1009333.s003.pdf]

		A-suppression*		A-replacement or B†		
Parameter	Description	Haplo-sufficient	Haplo-insufficient	Neutral	Haplo-sufficient	Haplo-insufficient
s_I	Fitness cost for target site disruption by insertion of construct	1	1	0	0	0
h_I	Dominance coefficient for target site disruption by insertion of construct	0	1	0	0	0
s_R	Fitness cost for target site disruption by resistant allele	1	1	0	1	1
h_R	Dominance coefficient for target site disruption by resistant allele	0	1	0	0	1
s_{IR}	Fitness cost for heterozygotes (construct/resistant)	1	1	0	0	1

s_I, s_R and s_{IR} are selection coefficients associated with host gene disruption and h_I and h_R are dominance coefficients. At locus A in suppression double drives, fitness costs for disruption of function at the target site can occur either by insertion of the construct (I) or by end-joining that produces non-functional cleavage resistance (R), resulting in recessive or dominant female-specific lethality for haplo-sufficient or -insufficient genes respectively. At locus A in replacement double drives, or at the differentiated locus B in either suppression or replacement drives, fitness costs for disruption of the target site by construct insertion are assumed to be zero (e.g., by having the construct contain a recoded version of the target gene). Where the target site is a haplo-sufficient or -insufficient essential gene, non-functional cleavage-resistant mutants are expected to cause recessive or dominant lethality respectively, affecting in both males and females.

* Fitness costs are female-specific.

† Fitness costs apply to both males and females.
