# Supplementary figures and images for: Double drives and private alleles for localised population genetic control
Source: PLoS Genet. 2021 Mar 23;17(3):e1009333. doi: 10.1371/journal.pgen.1009333 (PMC8018619; doi:10.1371/journal.pgen.1009333)

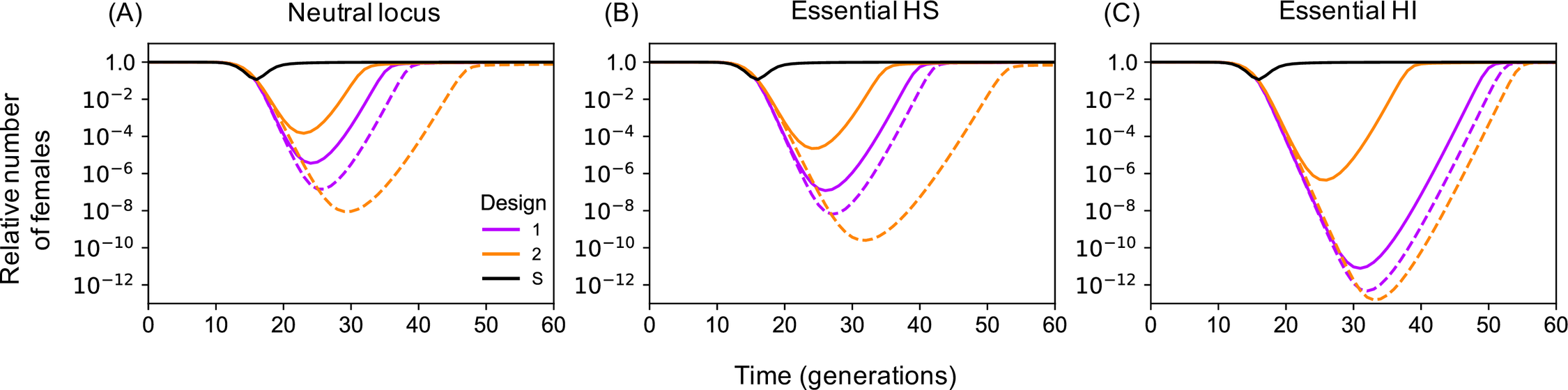

Supplement: S1 Fig — Solid lines are for where ⍺ and β are unlinked and dashed lines for where they are linked (r = 0.01). Shown are the cases where β is inserted as a neutral insertion into (A) a neutral locus, (B) an essential haplo-sufficient gene or (C) an essential haplo-insufficient gene. Shown for comparison is a time course for a single drive targeting a haplo-sufficient female-specific viability gene (S). (TIF) [file pgen.1009333.s004.tif]

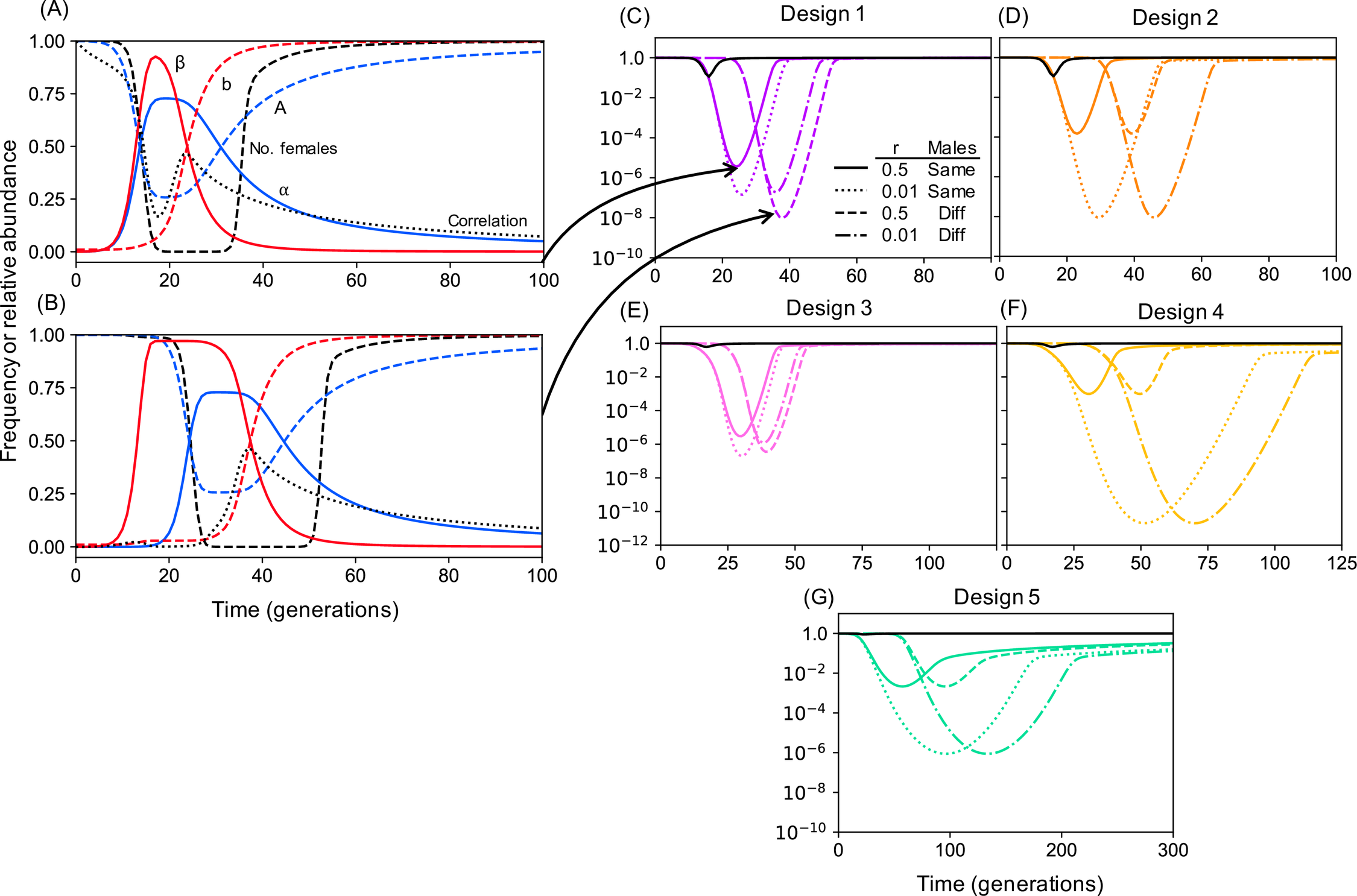

Supplement: S2 Fig — Comparison of dynamics for Design 1 when the constructs are unlinked and are released in the same (A) or in different (B) males. If the constructs are released in separate males the initial correlation between ⍺ and β (black dotted line) is negative, allowing β (solid red line) to increase to a higher frequency than if released in the same males as ⍺ where it experiences higher fitness costs. Consequently ⍺ (solid blue line) is retained at high frequency in the population for longer resulting in a greater reduction in relative number of females, though there is a longer delay between release and impact. (C-G) Timecourse for the relative number of females over time for Designs 1–5 where constructs are unlinked and released in the same males (solid lines), linked and released in the same males (dotted lined), unlinked and released in different males (dashed lines) or linked and released in different males (dot-dashed). Pre-existing resistance is assumed to be 1% (C, D), 20% (E, F) and 50% (G). For Designs 2, 4 and 5 (D, F, G) separate releases only delay the impact because β cannot increase in frequency autonomously, whereas for Designs 1 and 3 separate releases can give a larger (though still delayed) impact when constructs are unlinked, but not when they are closely linked. Shown for comparison is a time course for a single drive targeting a female-specific viability gene with the same level of pre-existing resistance (1%, 20%, or 50%; black solid lines). (TIF) [file pgen.1009333.s005.tif]

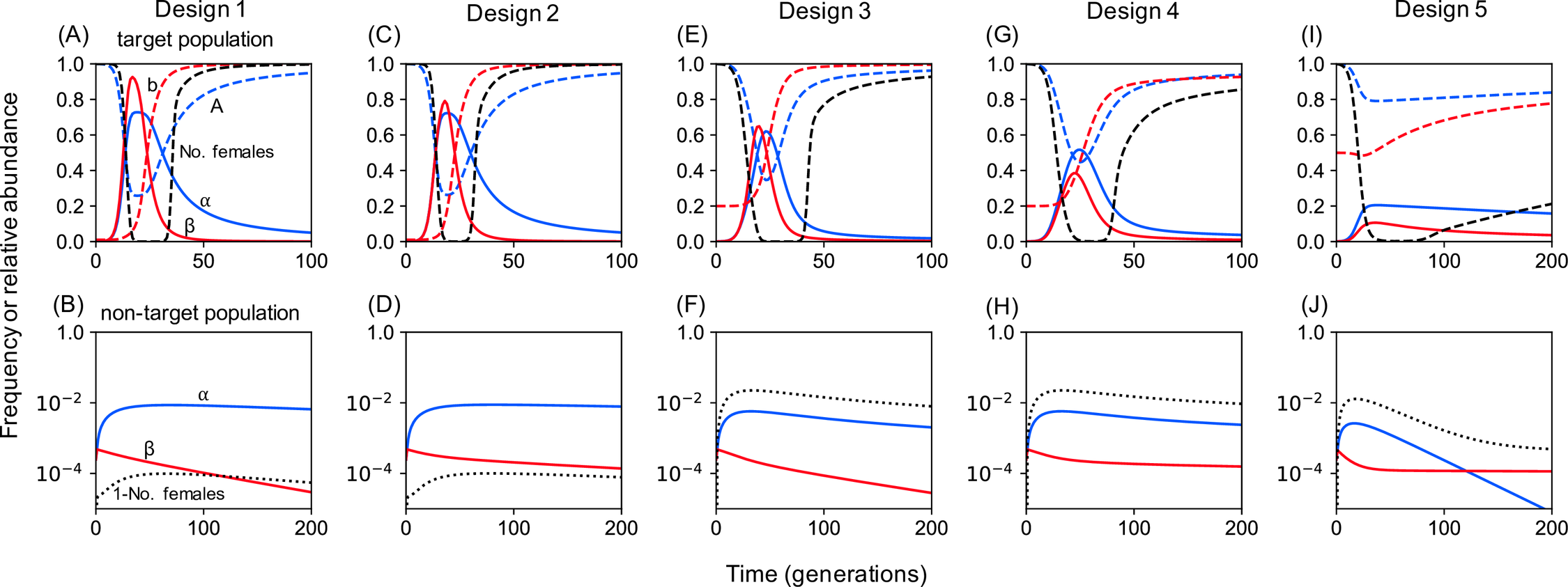

Supplement: S3 Fig — Design 1 (A, B) and 2 (B, C) assuming 1% and 100% pre-existing resistance in target and non-target populations. Design 3 (E, F) and 4 (G, H) assuming 20% and 100% pre-existing resistance in target and non-target populations. Design 5 (I, J) assuming 50% and 100% pre-existing resistance in target and non-target populations respectively. Plots for Designs 1, 3, and 5 are the same as in the main text, and presented here to facilitate comparisons. (TIF) [file pgen.1009333.s006.tif]

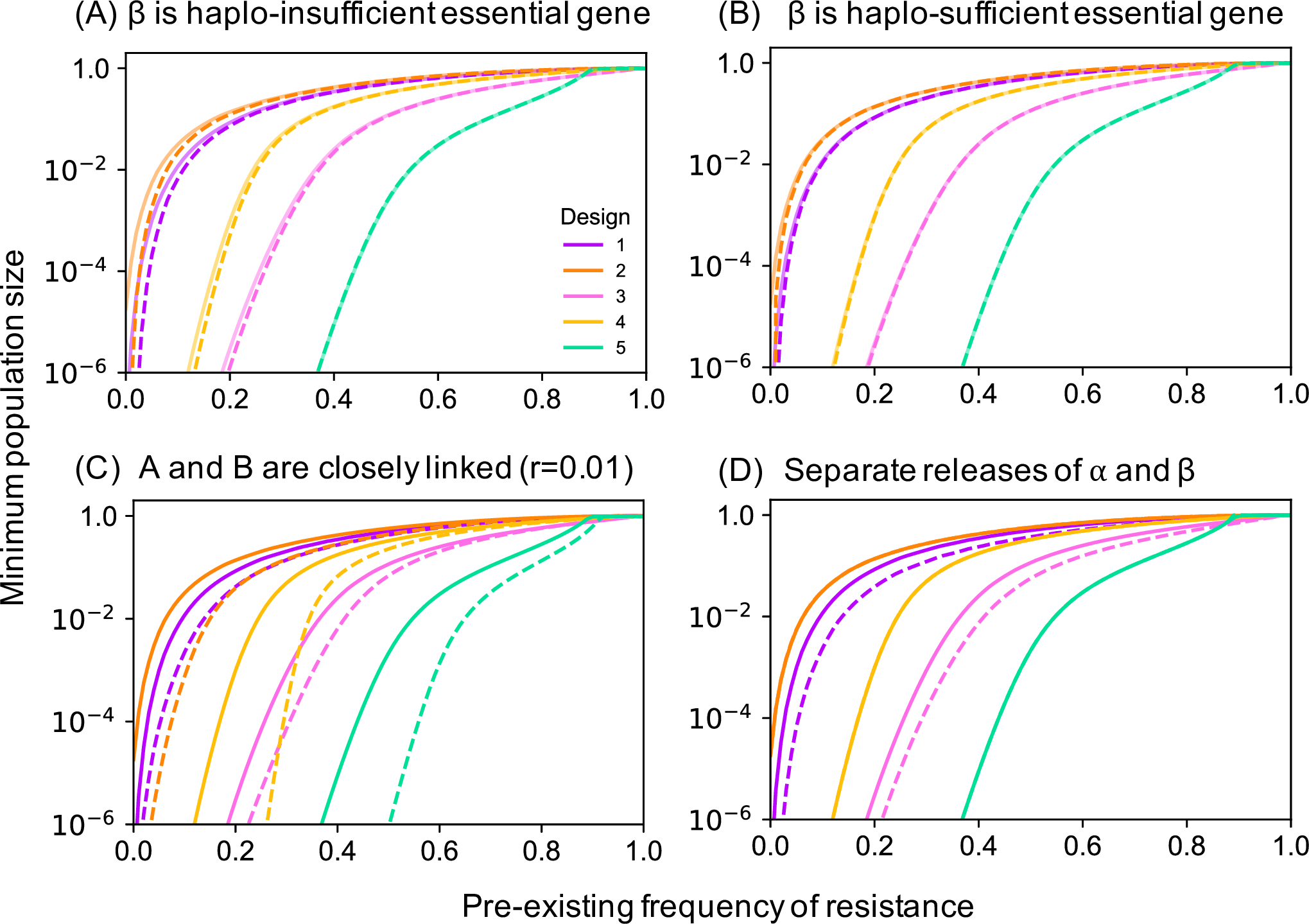

Supplement: S4 Fig — Solid lines are for baseline conditions (⍺ and β are released in the same males, β is in a neutral locus, and loci are unlinked), and are the same in each panel. Dashed lines are for variants where (A) β is inserted as a neutral insertion into an essential haplo-insufficient gene, (B) β is inserted as a neutral insertion into an essential haplo-sufficient gene, (C) loci are linked (r = 0.01), and (D) ⍺ and β are released in separate males, holding all other properties at baseline. (TIF) [file pgen.1009333.s007.tif]

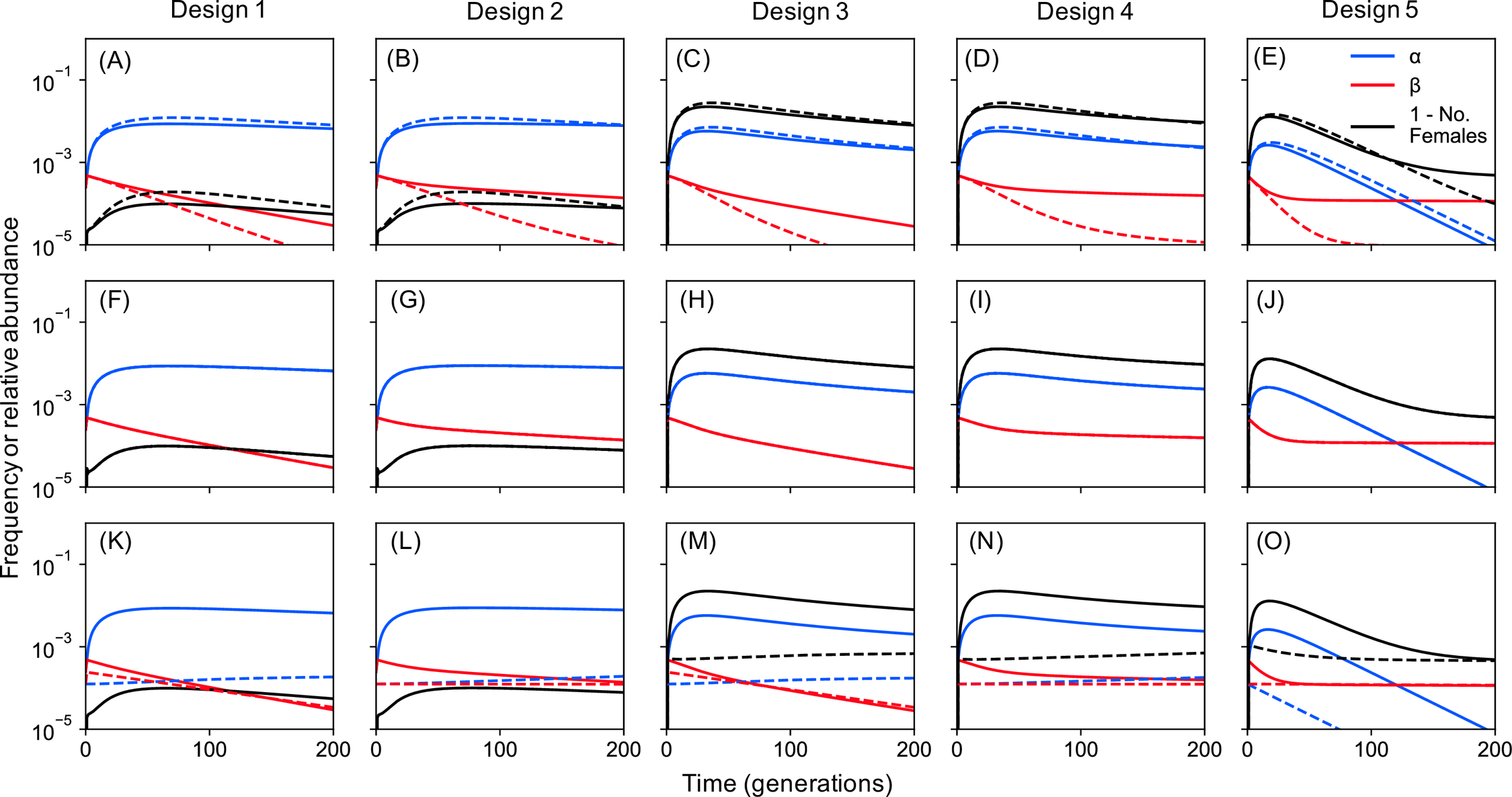

Supplement: S5 Fig — (A-E) ⍺ and β are unlinked (solid lines) or linked (dashed lines). (F-J) β is inserted into a neutral site (solid lines). Note that the effect of inserting β as a neutral insertion into a haplo-sufficient or haplo-insufficient essential gene would be virtually indistinguishable from the solid lines. (K-O) ⍺ and β are released in the same males (solid lines) or different males (dashed lines). (TIF) [file pgen.1009333.s008.tif]

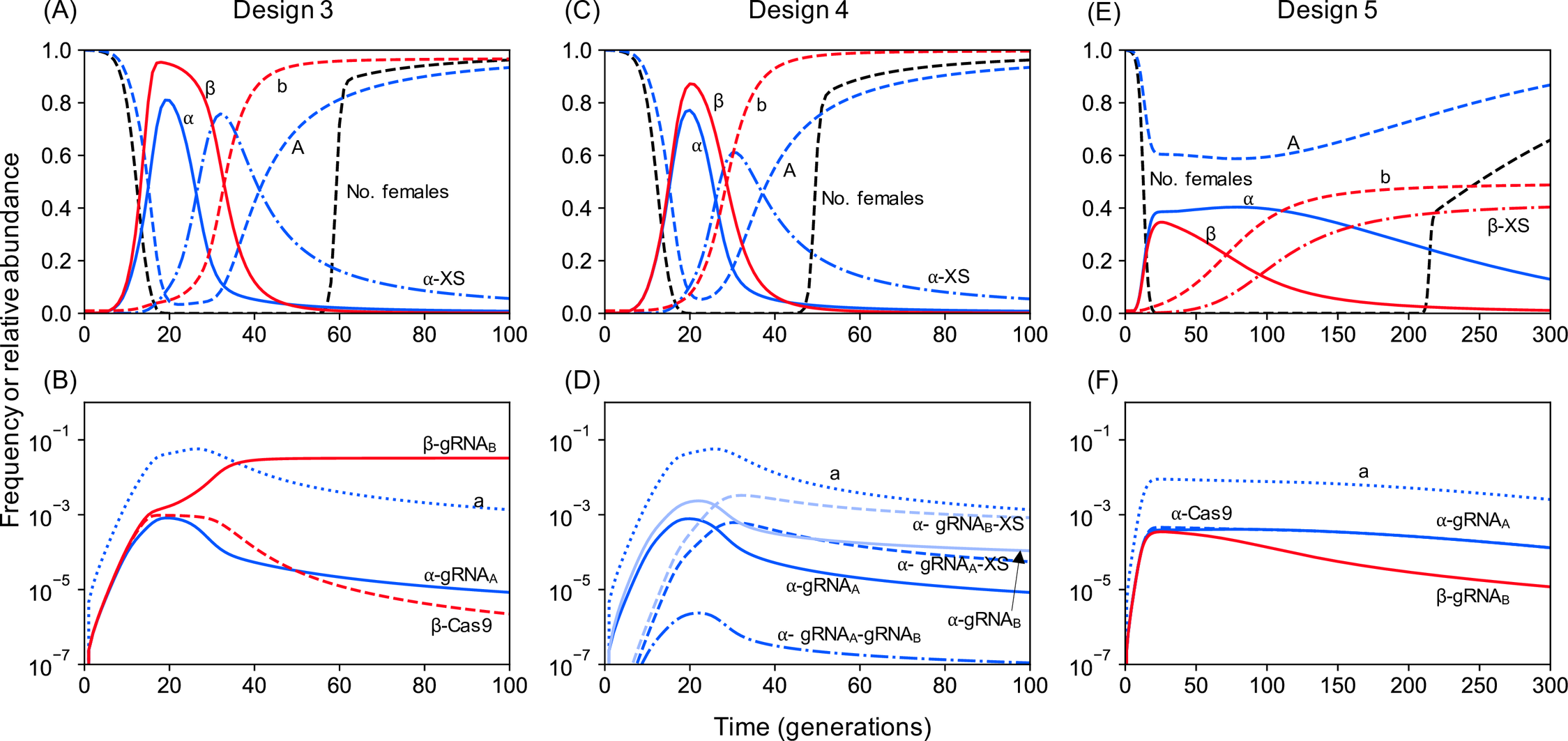

Supplement: S6 Fig — For each design, the intact constructs (⍺, blue solid lines and β, red solid lines) increase in frequency together, causing the relative number of females (black dashed lines) to decline. For designs 3 and 4, loss-of-function mutations at the X-shredder (⍺-XS, blue dashed-dotted lines) are selected for, replacing ⍺ (A, C). Since ⍺-XS is identical to ⍺ in designs 1 and 2, the construct continues to reduce the relative number of females. If the population is not eliminated, β is eventually replaced by the resistant b allele and ⍺-XS is replaced by the wild-type A allele, allowing the population to recover. For design 5, loss-of-function mutations at the X-shredder (β-XS, red dashed-dotted lines) are also selected for, but increase in frequency more slowly than the ⍺-XS allele in Designs 3 and 4, resulting in the intact β construct persisting for longer. For all designs, loss-of-function mutations at each of the other components (Cas-9, gRNAA and gRNAB) remain at low frequency, having negligible impact on the efficacy of the designs (B, D, F). Note that these results are not directly comparable to Figs 2 and S3 due to differing pre-existing resistance frequencies. (TIF) [file pgen.1009333.s009.tif]

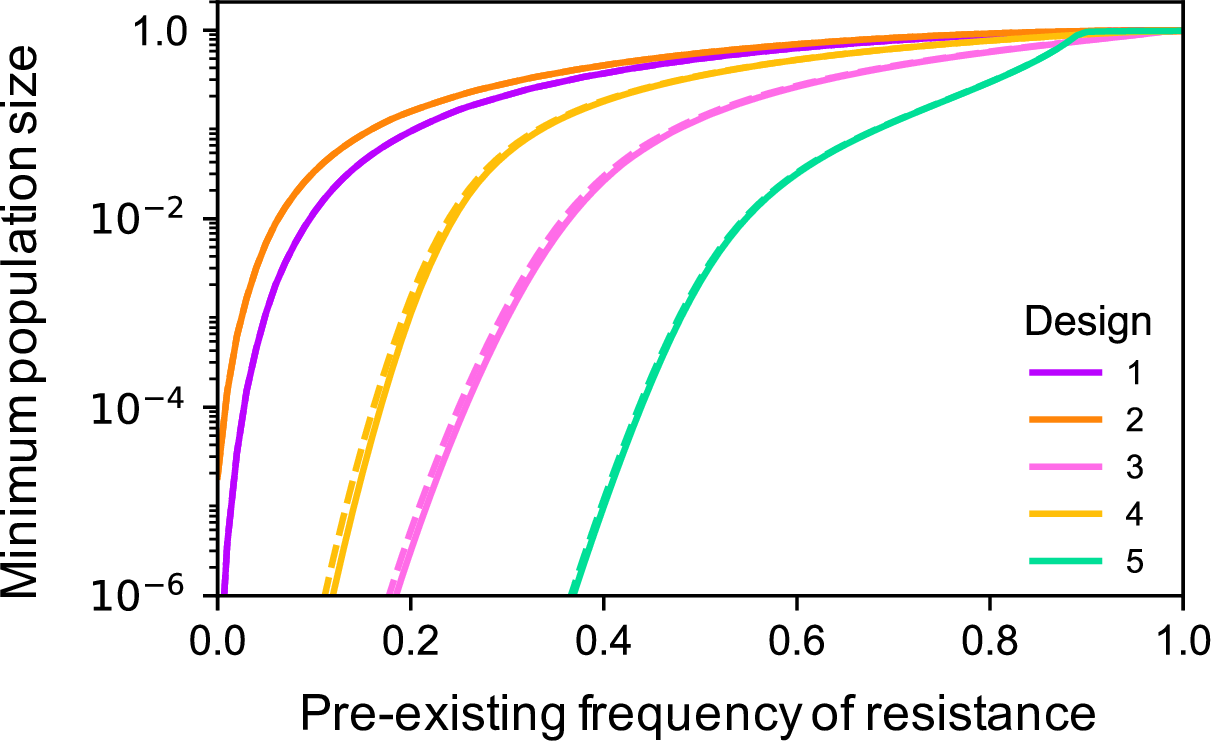

Supplement: S7 Fig — Solid lines are for baseline conditions where constructs remain intact after release, while dashed lines are for homing-associated loss-of-function mutations occurring at each component of each construct with probability 10e-4. (TIF) [file pgen.1009333.s010.tif]
