# Supplementary material for: Double drives and private alleles for localised population genetic control
Source: PLoS Genet. 2021 Mar 23;17(3):e1009333. doi: 10.1371/journal.pgen.1009333 (PMC8018619; doi:10.1371/journal.pgen.1009333)
Supplement: Attachment — Submitted filename: WillisandBurt_response_to_reviewers.pdf [file pgen.1009333.s011.pdf]

Dear Authors,

We now have three detailed reviews of your manuscript. All of the reviewers have positive comments on your work. There is, however, a real need to revise the text to make the concepts and design more accessible to the general reader of PLOS Genetics. The reviewers provide some guidance. The manuscript is accepted with "minor revisions" because no further analyses are needed, but substantially improved accessibility of the material is a necessary condition for acceptance. This includes accessibility of the figures. One review had some concerns about whether this paper really broke new ground and another commented that it would have been useful (as well as traditional) in the introduction to mention other related work and concepts--the introduction could demonstrate the novelty of your ideas by presenting what came before them. One reviewer suggested further analyses to examine spatial structure, but also recognized that this could be beyond the scope of an initial paper--I agree. Finally, one reviewer requested that the model code be made available to others. This makes sense to me.

We thank the editors and reviewers for encouraging us to more fully describe our ideas and results, which we have endeavoured to do. Please see below for more specific replies.

Your revision should be accompanied with a point by point response to each reviewer comm

In addition we ask that you:
1) Provide a detailed list of your responses to the review comments and a description of the changes you have made in the manuscript.
2) Upload a Striking Image with a corresponding caption to accompany your manuscript if one is available (either a new image or an existing one from within your manuscript). If this image is judged to be suitable, it may be featured on our website. Images should ideally be high resolution, eye-catching, single panel square images. For examples, please browse our archive. If your image is from someone other than yourself, please ensure that the artist has read and agreed to the terms and conditions of the Creative Commons Attribution License. Note: we cannot publish copyrighted images.

We hope to receive your revised manuscript within the next 30 days. If you anticipate any delay in its return, we would ask you to let us know the expected resubmission date by email to plosgenetics@plos.org.

If present, accompanying reviewer attachments should be included with this email; please notify the journal office if any appear to be missing. They will also be available for download from the link below. You can use this link to log into the system when you are ready to submit a revised version, having first consulted our Submission Checklist.

While revising your submission, please upload your figure files to the Preflight Analysis and Conversion Engine (PACE) digital diagnostic tool. PACE helps ensure that figures meet PLOS requirements. To use PACE, you must first register as a user. Then, login and

navigate to the UPLOAD tab, where you will find detailed instructions on how to use the tool. If you encounter any issues or have any questions when using PACE, please email us at figures@plos.org.

Please be aware that our data availability policy requires that all numerical data underlying graphs or summary statistics are included with the submission, and you will need to provide this upon resubmission if not already present. In addition, we do not permit the inclusion of phrases such as "data not shown" or "unpublished results" in manuscripts. All points should be backed up by data provided with the submission.

PLOS has incorporated Similarity Check, powered by iThenticate, into its journal-wide submission system in order to screen submitted content for originality before publication. Each PLOS journal undertakes screening on a proportion of submitted articles. You will be contacted if needed following the screening process.

To resubmit, you will need to go to the link below and 'Revise Submission' in the 'Submissions Needing Revision' folder.

Please let us know if you have any questions while making these revisions.

Yours sincerely,

Fred Gould
Guest Editor
PLOS Genetics

Gregory Copenhaver
Editor-in-Chief
PLOS Genetics

Reviewer #1:

Synthetic gene drives have considerable potential for landscape-scale suppression of wild populations that carry disease agents (such as malaria) or cause significant environmental or agricultural damage (e.g. invasive pests). While there is considerable excitement about the prospect of gene drive deployment, there are also legitimate concerns about the potential impact of gene drive leakage into non-target populations. Recent studies have indicated that one possible strategy for target-population specific control is to exploit genetic differences in target and non-target populations – for example Sudweeks et al investigated if “private alleles” and be used for population suppression via single gene drive strategies. Such approaches are very sensitive to pre-existing resistance alleles – essentially the private allele needs to be completely fixed in the target population (but low levels in the non-target population can be tolerated). Here, Willis and Burt investigate if an alternative (more likely?) genetic architecture can be exploited – whereby the differentiated allele is present in the target population at high frequency (but not completely fixed) and not in the non-target population. Various double drives strategies are examined using a simple deterministic modelling approach.

Some scenarios, even with relatively limited differentiation in the target population (e.g. 50% pre-resistance, Design 5), show quite remarkable suppression dynamics. Drive designs are realistic (based on existing studies at least in insects) and modelling parameters based on experimental data in An. Gambiae (noting that homing drives seem particularly efficient in Anopheles). It would be really interesting to see how the strategies perform with more realistic stochastic, individual-based (spatial) modelling but I accept their argument that this "first-pass" analysis.

The manuscript is clearly written, logically presented and is a significant body of work. I do not have any major concerns.

We thank the referee for their positive comments.

The following points should be addressed.

1. Line 201-5 This is confusing – the text mentions "…killing heterozygous female (Fig 1 Designs 3 and 4)" – but Fig 1 indicates design 3 and 4 are haplosufficient (only design 5 is haploinsufficient). Further the Simoni et al [27] referenced gene drive is inserted into a haplosufficient gene.

Yes this was a mistake, it should have read "homozygous female". We have now corrected it (line 259 of the "tracked changes" document).

2. Design 5 is a little unclear. The alpha locus drives autonomously in males (line 210) – this should be indicated in Fig. 1. Given there is only one Cas9 source (from the alpha locus) – then locus B should also be homing only in males. I cant see this explicitly mentioned and want to confirm this is what was modelled.

Yes that is correct and we have now included a sentence in the text making this explicit (lines 273-274).

3. Very tight linkage between the alpha and beta loci could be confounded by the generation of large deletions (generated by end-joining after simultaneous DBSs) – this should be acknowledged in the Discussion.

Yes, we now make clear in the discussion our assumption that the two constructs are not so close that they interact in this way (lines 483-489).

4. Line 125-6 Some explanation should be provided regarding the strategy whereby functional resistance alleles will be avoided.

We agree and have now indicated that this could be achieved by targeting essential sequences or using multiple gRNAs and have given example references (lines 144-146).

5. At lines 145-6, given an essential gene is not used in this context, it should be made clearer how the population rebound is occurring i.e. end-joining indels versus selection for the 1% non-target alleles that are present at the outset.

Selection is happening on both end-joining mutants and pre-existing resistant alleles, so both contribute, and we now make this explicit (lines 171-177).

6. The significance of including the An. coluzzii data (Fig. 7) should be make clearer. It seems only An. gambiae is relevant. Also, why include the "uncertain" population in the legend?

They were included because there can be widespread hybridisation between the species and the distinction between them breaks down in some locations, and therefore a homing construct released in one could well transfer to the other, if there is the recognition sequence. This is now made clear in the S1 Text Supplemental methods (lines 1150-1151). However, the distinction is not particularly relevant to the points we are making in the main text and, for simplicity, we now use the same symbol in Fig 7 for all populations used in the comparison.

7. Fig 7 shows a "proof-of-concept" analysis for the existence of "private allele" PAM sites in hypothetical target (island) versus mainland populations. Although the limited analysis is probably sufficient to make their point it would be interesting to know location of the PAMs, (i.e. the types of genes they reside in, particularly given that some strategies rely on an essential gene for the Beta locus), predicted off-target profile and on-target activity of their cognate protospacer sequences.

We agree that these are interesting follow-up questions raised by our proof-of-concept analysis, and there are others too, but answering them is beyond the scope of this current paper.

8. Line 358 A small caveat here that is hinted at but could be made more obvious is that the X-shredder could not be Cas9- based (if the homing constructs also use Cas9) – Cas9 is used for X-shredding in ref 43 and 44 but of course I-PpoI has been developed in An. Gambiae.

Yes there are constraints but the details are complicated. For example the X-shredder and homing could both be Cas9 based if there was tissue specific expression of the gRNAs. We have now added a sentence to make more explicit the issue (lines 496-500).

9. Given that homing rates in other species are generally lower and Anopheles, it would be interesting to see an analysis of a high performing drive strategy with cleavage/indel/homing rates from another well characterised insect – e.g. Drosophila melanogaster. Indeed, a sensitivity analysis for a high performing strategy would enhance the paper.

We have performed a sensitivity analysis for two fitness costs for the five designs (Fig 5) and any more is beyond the scope of this paper.

10. Line 78 The phrase "in the computer" is a touch awkward.

We believe the phrase adds useful detail to the sentence and therefore have left it in.

11. Line 197 Reference Sup Fig S4 here.

Actually, the point is best shown in Fig 4, and we now cite that (line 254).

12. Line 431-2 Reference table S1 here.

Done (line 596)

13. What does "alpha-cargo" signify in Fig 6B?

It was mean to signify "alpha construct minus the cargo", but we acknowledge this was confusing (also to Referee 2). We have now added a legend to the figure, and refer to the line as "cargoless alpha construct", and have added a sentence to the caption: "cargoless α construct" refers to α constructs that have lost their cargo gene."

Reviewer #2:

This is an interesting paper in which methods (double drive) for local and transient (assuming extinction does not occur) population suppression, and modification, are described. Several other multi-component HEG-based systems have been previously described for modification. These are often somewhat baroque and have a number of requirements that may not be met in the real world in terms of target site conservation, etc. Other non-HEG-systems have also been proposed. This paper rises above this earlier work because it cleverly leverages the features of HEGs (in anopheles mosquitoes) that are known to work, in ways that make success likely. In particular, it leverages the fact of resistance allele formation in ways that are uniquely positive, rather than negative, as well as the previously reported ability to target the dsx locus without resistance allele formation, and to bias sex ratio using autosomal X shredders. In short, while this is a purely modeling paper, there is little doubt that multiple of the strategies described can be successfully implemented in Anopheles, and with some biological luck, elsewhere.

Thank you for your positive comments.

The simplest version of the idea is clear. An alpha element is designed to insert into and disrupt the female-specific functions of dsx, resulting ultimately in unfit homozygous females and fit males. This element is non-autonomous, containing either gRNAs or Cas9, but not both. A second element, beta, is inserted at a neutral locus that is enriched within the target population, and rare outside it. Beta is autonomous, and complements the missing alpha function, resulting in homing of alpha when both elements are present in the same individual. What makes this design (and related designs in which there is cross-complementation of various sorts) interesting is the fact that failure of the beta element to spread to all versions of its target site is guaranteed, and does not disrupt the intent. Beta's job is simply to increase in frequency enough to push alpha towards fixation, at which point population suppression ensues. Beta can either target a private allele, or in a more general version of the system, simply a neutral locus, with the inevitable appearance of resistant alleles ultimately limiting the spread of it and alpha.

That is an interesting point: we have all along been focussed on the use of sequence differences to prevent spatial (or taxonomic) spread, and not considered the possible use for transient control when, for whatever reason, there is no concern about spatial spread, and we now describe this possibility in general terms in the discussion (lines 456-462). Using an undifferentiated neutral locus for beta would not give spatial control, but there may nonetheless be some specific use cases.

Thus, the key here is that with the designs proposed the authors are able to take advantage of what we (the field) already know how to do really, really, well, which is to build a HEG that fails due to resistance allele formation or the presence of pre-existing polymorphisms that are resistant alleles. The designs are plausible, because they utilize the known: consistent targeting of dsx, inconsistent targeting of pretty much any other site, either due to pre-existing polymorphisms, or new resistant allele formation, and X shredding-based sex ratio bias.

My one specific request is that the authors provide the code on github for their work, along with an explanation of how to use it. Referencing back to a paper from 2008, which also does not provide code, does not provide sufficient guidance to understand and explore the many variables, loci and alleles worked with in the current manuscript. It is necessary that others be able to not only read the text, but also work with tools that allowed its creation. In the absence of these methods the paper is hard to explore, and it is not possible to test other scenarios. The details of how the model was implemented are undoubtedly very interesting. It clearly involved a lot of work (which remains invisible in the background otherwise), as it involves many alleles at multiple loci, shredding, homing and recombination. One hopes that general models such as this that can handle a lot contribute to the field eventually adopting a coherent modeling platform rather than a series of lab-specific one-offs.

The code has now been put on github and a link added to the methods (line 622). The code is indeed useful for addressing many other interesting questions and we are in the process of doing so.

The manuscript is very dense and almost always asks the reader to already be intimately familiar with the behavior of related systems. The text describing the different designs and outcomes reads a bit like a math textbook. There is no walk through for the uninitiated, and sections typically end with a statement of outcome, in much the way a math text book would end a proof with the phrase "from inspection it can now be inferred that...", which then leads to a figure and a turn to a new chapter.

Or to put it another way, in reading and re-reading the text and very dense figures (which rarely or never guide the reader through the data), I was reminded of the first line from Ludwig Wittgenstein's Tractatus, Logico Philosophicus: "This book will perhaps only be understood by those who have themselves already thought the thoughts which are expressed in it—or similar thoughts."

I would like to strongly suggest that the authors make more of an effort to walk readers through the designs and how they work. The figures are very dense and key features of the panels are never discussed. The figures are simply presented, along with very minimal text, and the authors then move on to the next section, with the implicit

understanding that the reader will somehow muddle through.

Other suggestions are below. Many of these also relate in one way or another to expanding discussion to make the work more accessible to non-specialists. The ideas presented are really beautiful and plausible, and I just think that they can be made more accessible in ways that will allow others to better intuit the forces at work and how the ideas presented could be extended further. The manuscript is not overly long, and is online only in any case. If the authors feel strongly for some (compelling) reason that they want to keep the text tight and telegraphic they could also consider an extended supplementary discussion of the various systems. A nice example of this is the remarkably complete supplemental notes file provided in another article from this group "How driving endonuclease genes can be used to combat pests and disease vectors"

We thank the referee for encouraging us to expand on our thoughts and results, and we have done so at many places throughout the text, as the "tracked changes" version shows.

In the introduction the authors might consider introducing other double drives from the literature. This would allow the authors then to begin drawing distinctions between prior goals and their own, and how their constructs etc therefore differ. See related comments on discussion.

We think it is useful to keep the Introduction focussed on the objective at hand – localised population genetic control – rather than review all previously proposed two-construct strategies with their various rationales, but we have expanded the description of prior work on localised control (lines 90-95). "Double drives" is a new term introduced in this paper, and we consider its definition best left to the Discussion, after the reader has a clear example in mind of what is meant. We have also expanded the description of previously described double drives in the Discussion, referring to their different rationales (lines 444-446).

Its also a bit hard, in figures like 2 and other similar figures, to keep track of what all the lines mean. It requires going back and forth between the figure legend and Figure 1. It would be nice to introduce these a bit more visually, perhaps in a box at the top of figure 2. And again, none of this is discussed in the text.

We have now added a legend to figures 2 and 6B, and edited the captions for increased clarity.

It might be nice if the authors would note at some point that the idea of using linkage to increase drive strength has been noted and explored to some extent in other systems.

Agreed, we now do this both when we first introduce the idea of variable linkage (lines 196-204) and in the discussion, where we compare our results to those recently published by Oberhofer et al. (2021) (lines 465-483).

In figure 3 it would be good to walk the reader a little bit through the significance of targeting a haploisufficient locus that manifests phenotypes in particular

sexes/genotypes. Again, this is one of those points in the text where the reader really needs to have a deep familiarity with the prior literature in order to understand how and when this manifests itself, and how this contributes to suppression while still allowing strong drive.

We have expanded the discussion of this point (lines 210-212)

In the legend for figure 3 it is not immediately clear what is going on with the single locus drive and why it works so much less well. Since a single locus drive is presumably inserted into the dsx locus, which thus far does not accumulate resistance alleles, what causes the failure? The legend makes no mention of these or other potentially important variables. Is there a resistance allele in there somewhere that is not mentioned? If so, why is it present in single locus dsx, but not the alpha locus of the two locus versions? Legends should include all the relevant variables at work.

Agreed, and we have added an explanation both to the legend and main text to clarify (lines 246-247 and 225-229).

In line 200 it would be nice to explain in a bit more detail why the x shredder kills heterozygous females. When is it expressed and how does this still allow rapid and strong drive

This was incorrectly phrased, we have now clarified that it is alpha doing the killing, not the x-shredder, and it is homozygous females that die, not heterozygous (lines 258-259).

In lines 211 onwards this is the first place where the authors make an attempt to walk the reader a bit through how the system, a particularly complicated one, does its job.

Thank you, we have now tried to replicate this for the other designs.

Line 239. This is an odd reference for the idea that hegs have reduced replication fidelity. While the referenced construct does have reduced stability due to the presence of repeats, this is not the same thing as the reduced fidelity that may be associated with homing per se. Is it appropriate to also reference some of the evidence suggesting HR repair of a ds break may lead to higher frequency of mutation, or do you just mean to focus on the repetitive components, gRNAs, if multiple are used? Though if dsx is being targeted with a single gRNA then it is not clear there is a repetitive element.

We have added two more references (lines 307).

For Fig 6 I don't quite understand the lines in panel B. In particular why is the frequency of alpha different from that of alpha-cargo. The alpha construct contains the cargo, so shouldn't they be the same? This figure is also one where a bit more captioning in the figure itself would help the reader. Finally, If alpha-cargo is the entity of interest, wouldn't it make more sense to show its genotype frequency rather than allele frequency, or at least both? The allele frequency appears to saturate at about 65-70%, but it is unclear what this means in terms of carrier frequency.

Alpha-cargo was meant to refer to the alpha construct which has lost its cargo (alpha minus cargo). We acknowledge this was a confusing label and have changed it to "cargoless alpha construct" and further clarified its meaning in the caption.

In Fig. 6C the non-target population seems to perhaps rise to a significant number, maybe 30%. It is hard to know what the number is since its a log scale. It would be useful here and perhaps a few other places to provide actual numbers so the reader can evaluate this, since non-target population effects are the topic that tends to get folks excited.

There was an inconsistency here in that the main text refers to the non-target population being fixed for the resistant allele (as we also assume for all the population suppression simulations), but Fig 6C referred to only 90% pre-existing resistance (i.e., 10% cleavable alleles). This is confusing and we have replaced this figure with simulations of non-target populations fixed for the resistant allele, to be consistent with the rest of the paper. For information, the maximum frequency reached under the old assumption of 10% pre-existing cleavable alleles was 0.057 and 0.047 for design 1r and 2r respectively.

The data presented on private allele frequencies is intriguing, and the authors are appropriately cautious about its interpretation given the limited data available.

In lines 358-353 in the discussion, the authors bring up, for the first time, other two locus systems. Some, such as myself, would have placed this and more in a manuscript introduction that put the current work on two locus systems into a historical context that discussed what had come before, including other two locus homing systems. The authors have chosen not to do that, or provide any more than a quick reference nod to this and other literature in their discussion. It is an interesting strategy, as it keeps the readers attention squarely focused on the authors' thoughts and accomplishments, while minimizing any mental clutter from the literature that might interfere with this task. That said, while the prior literature is not discussed, its existence is noted, and thus represents a stylistic choice that is acceptable.

Thank-you. We do believe in keeping the readers' thoughts resolutely focused on the specific design objective of the paper, particularly in the Introduction. Our focus is on using genetic differentiation to achieve localised population genetic control, and, as far as we are aware, there is only really one previous paper on this topic, and we describe it in detail and compare our results with theirs throughout the paper. That said, it is important to acknowledge and cite papers that propose methods for localised control by other means (essentially, by requiring large releases), and to relate our designs to designs proposed by others for other objectives. We have expanded somewhat our description of previous two-locus strategies and the rationales for which they have been proposed, both in the Introduction (lines 90-95) and the Discussion (lines 444-446), while hopefully not distracting from our main focus. There will be a time and place for a detailed review of two-locus systems, but suspect there is much more to be discovered first, and in any case that is beyond the scope of this paper.

In line 363 the authors discuss the idea of adding extra gRNAs to the alpha construct. They say this will increase the load, but they dont explain how this will work. Can they

do this, briefly? Spitting off LOF mutations (or HEGs?) at independently segregating loci does this because......? Again, the diligent reader can at some point come up with a plausible scenario, but the authors could guide them to this point directly in a few lines. The two references provided may not be the most relevant. One is an old paper that talks about several uncloned maternal effect mutants and their effects on embryo development, and the other discusses roles of fruitless in males, not females. Is there something more relevant based on cloned genes with known behavior that could be cited? In any case, the key point is to walk the reader through how these alleles bring about increased load while still allowing drive.

Agreed, and we have added some explanation (lines 506-511). Also, there was a glitch with the references which has now been corrected to refer to previous suggestions of adding nucleases to create LOF mutations as a way to produce load.

On line 144 the authors refer to dominant fitness costs. Can they clarify that this does not mean (I assume) completely dominant, as in heterozygote fitness cost = homozygote fitness cost. If it does mean completely dominant can they explain the basis for this, as I would normally imaging fitness costs to be additive in some sense. Are they meaning to imply that off target cleavage effects are 100% regardless of the dosage? That might explain it.

We do mean completely dominant, as in heterozygous fitness cost = homozygous fitness cost, on the thinking that fitness effects due to off-target cleavage will be the same regardless of dosage, and similarly we assume the effect of the cargo on female fitness is dominant, matching the standard assumption that effects on vector competence are dominant (i.e., one copy of the construct preventing transmission). We now make this more explicit (lines 614-615).

Reviewer #3:

Willis and Burt present a simulation study of gene drive approaches that could potentially be localized to specific target populations. They focus on a combination of two basic concepts: two-locus drives (aka “double drives”) and the targeting of “private” alleles. The key idea is that there is a division of labor between the constructs at the two loci, with the first responsible for the desired impact (population suppression or replacement) and the second for the localization, achieved through the targeting of a private allele. The first construct can only drive when the second is present. In the target population, both constructs are therefore expected to drive, whereas they would resemble a split drive in non-target populations. The paper conducts computer simulations of a number of different such strategies.

This is a well-written paper on an interesting topic. It is definitely more plausible that a real-world use case of a gene drive might exist for a type of drive that can be localized, as compared to standard homing drives that have the potential to spread around the world. The inclusion of an analysis of actual polymorphism data in a mosquito species to see how common the required “private” alleles are observed is quite interesting as well. I think we generally expect these sorts of sequence variations to be present

between differentiated populations, and I wouldn't say that it is critical that a modeling paper include this kind of analysis, but it's interesting to see.

Thank you for your positive comments.

My main comment is concerned with the extent to which this study provides an advancement over previous studies that have already provided a proof-of-principle of double drives achieving localization by targeting private alleles (notably, the recent study by Sudweeks et al). The authors of the current study are very clear that their study is still intended to serve as a proof-principle, rather than an assessment of an actual potential application, which would certainly require more detailed and realistic modeling to be meaningful (although they do study specific populations of An. gambiae in their analysis of polymorphism data). As such, the current study appears to re-tread some conceptual ground that has already been explored in previous studies. Obviously, there are some differences between the specific drive designs studied in their paper and Sudweeks et al. The latter study also focused on sequences that are fixed in the target population, but absent or at low frequency in the non-target population (the opposite of the current study). Whether these differences constitute enough advancement for the journal is a subjective question I want to leave to the editor to decide.

We think there is some confusion here in that Sudweeks et al only consider single construct drives. This was an important first step and as noted by the authors their strategy relies on there being no pre-existing resistant alleles in the target population, which may not always be possible to find. Our paper is concerned with using double drives to localise effects, and we find in many ways that these designs can be substantially more effective than a single drive (which we model for comparison; black lines in Figures 3 and 6D). We have now made this explicit in the introduction (line 102) which, amongst other things, should make the distinction between Sudweeks et al and our work clearer.

Another (admittedly very general) concern is how confident we can be that even basic, qualitative conclusions from this study will ultimately hold in a real-world population, given the rather simplistic assumptions made by the modelling. In particular, it is unclear whether results from a panmictic population model still apply for realistic populations that are inherently spatially structured, for example, because they occupy a continuous landscape with limited dispersal. This can affect dynamics not only quantitatively, but can give rise to new qualitative phenomena that fundamentally change the outcome of a drive (e.g. resulting in a suppression drive failing to suppress a population). At the core of such new phenomena often lies the heterogeneity that can arise in spatial populations, which doesn't exist in panmictic models. I would expect that two-locus drive strategies would be particularly prone to such issues, as compared to single locus strategies. This is due to the assumption made by panmictic models that the frequency of allele combinations at the two loci is simply the product of their individual population frequencies. In a spatial population, however, the two alleles could spread at considerably different rates, and thus be present very heterogeneously. The panmictic model would then rely on fundamentally wrong predictions of how frequently such alleles would actually "meet" in an individual based on their overall population frequencies, and this could certainly affect the dynamics profoundly.

I admit that a comprehensive analysis of such questions would be open-ended and probably beyond the scope of the current paper. The authors do already discuss several other limitations of their model in the discussion section. Maybe a mention of the potential impact of spatial heterogeneity and limited dispersal could be added as well.

This is a good point, and we now discuss some potential differences between well mixed and spatially explicit models in the discussion (lines 572-676).

Minor comments:

The authors might consider reworking and extending the methods section. It is very short and seems a bit rushed compared to the rest of the paper. Some parameters are specified in this section, while not really enough detail is provided about what those parameters do. E.g. on line 428-430:

"juvenile survival is density dependent according to the Beverton-Holt model, which has two parameters, but since we report results in terms of relative population sizes, only one matters, the intrinsic rate of increase (Rm)."

Somehow this sentence simultaneously tells me what specific implementation the model uses, while telling me very little about that implementation, and tells me that there are two parameters, and then tells me that there is only one parameter. It also seems a little awkward that the PAM material and the modeling material are not in separate paragraphs. I am aware that these authors focused more attention on the supplemental methods section, but I feel that if there is going to be a short methods section in the main body, it should focus on giving a rough sketch of how the model works without going into unnecessary specifics, but ideally at least introducing all aspects of the model.

We agree the description of the density dependent model was confusing and have edited it, and have separated the description of the PAM analysis into its own paragraph. That said, we did not want to have two detailed descriptions of the methods, and the full details are in the SI. We would be happy to move this section to the main text if the editors would find this preferable.

2. I find the statement that their model is individual-based a bit misleading. The model used portions of the total starting number of individuals who are in a given state, and as the population declines, the simulation isn't tracking an actual simulated population size, but rather, a size relative to the equilibrium size. I'm sure this allows for simulations to be performed with great computational efficiency, and no doubt this type of model has its place in gene drive research. Still it is not truly an individual-based model, resulting in somewhat awkward situations such as that actual population elimination is never possible (as frequencies can become arbitrarily small but never truly reach zero).

We agree it would be misleading to describe our model as "individual-based". We do not think we did in the original submission, but the confusion may have arisen because we did refer to "individuals" in the methods. To avoid this we now use "genotypes".

3. The caption of Figure 2 seems to be missing some details (e.g. it doesn't state that the dashed lines indicate resistance). The figure caption could probably be simplified a lot if a legend were included with the figure. In general, I feel that several of the figures might benefit from having legends, especially because the same style of line means different things from figure to figure (e.g. in Figure 2, the dashed lines indicate resistance within the population, in Figure 3 the dashed lines indicate that construct β is inserted in a haplo-insufficient target). Including a legend would probably make the figures a fair bit easier to digest.

Agreed, and we have now added legends to Fig 2 and 6B with short descriptions. We have also tried to edit the captions for increased clarity.
